# Specific Localization of the Drosophila Telomere Transposon Proteins and RNAs, Give Insight in Their Behavior, Control and Telomere Biology in This Organism

**DOI:** 10.1371/journal.pone.0128573

**Published:** 2015-06-12

**Authors:** Elisenda López-Panadès, Elizabeth R. Gavis, Elena Casacuberta

**Affiliations:** 1 Department of Comparative Genomics, Institute of Evolutionary Biology, (CSIC-UPF), Barcelona, Spain; 2 Department of Molecular Biology, Princeton University, Princeton, NJ, United States of America; University of Otago, NEW ZEALAND

## Abstract

Drosophila telomeres constitute a remarkable exception to the telomerase mechanism. Although maintaining the same cytological and functional properties as telomerase maintain telomeres, Drosophila telomeres embed the telomere retrotransposons whose specific and highly regulated terminal transposition maintains the appropriate telomere length in this organism. Nevertheless, our current understanding of how the mechanism of the retrotransposon telomere works and which features are shared with the telomerase system is very limited. We report for the first time a detailed study of the localization of the main components that constitute the telomeres in Drosophila, *HeT-A* and *TART* RNAs and proteins. Our results in *wild type* and mutant strains reveal localizations of *HeT-A* Gag and *TART* Pol that give insight in the behavior of the telomere retrotransposons and their control. We find that *TART* Pol and *HeT-A* Gag only co-localize at the telomeres during the interphase of cells undergoing mitotic cycles. In addition, unexpected protein and RNA localizations with a well-defined pattern in cells such as the ovarian border cells and nurse cells, suggest possible strategies for the telomere transposons to reach the oocyte, and/or additional functions that might be important for the correct development of the organism. Finally, we have been able to visualize the telomere RNAs at different ovarian stages of development in *wild type* and mutant lines, demonstrating their presence in spite of being tightly regulated by the piRNA mechanism.

## Introduction

The telomere maintenance mechanism by telomerase is highly conserved among eukaryotes with the exception of some branches of the evolutionary tree. During evolution, telomerase was lost in Drosophila and likely in other dipterans. Different strategies that compensate for the lack of telomerase have been found in different insects [1], the best studied of these being Drosophila. In Drosophila, telomeres are elongated by the specialized and targeted transposition of three non-LTR retrotransposons, *HeT-A*, *TART* and *TAHRE* (from now on HTT array) [2], [3]. These three retrotransposons have established a symbiotic relationship with the host genome, inserting randomly as long head-to-tail arrays at the end of the chromosome when needed [3]. The mechanism by which the telomeres are elongated in Drosophila does not differ substantially from the one used by the telomerase ribonucleoprotein (RNP). In both cases, a template RNA is reverse transcribed onto the end of the chromosome, assisted by different proteins that are important for telomere targeting and regulation [4]. The specific steps of this mechanism in Drosophila are not yet known.

Several lines of evidence suggest that both the proteins and the RNAs encoded by the telomere retrotransposons are essential components of this mechanism (reviewed in [3]). The level of conservation of the genes encoded by the telomere retrotransposons, *HeT-A gag*, *TART gag* and *pol*, suggests the existence of a negative selective pressure [5]. Therefore, the proteins encoded by *HeT-A* and *TART* are likely necessary for their transposition and, as a consequence, for telomere elongation. Previous studies have shown that the Gag protein of *HeT-A* is essential for telomere targeting of the telomere RNP [6]. In contrast, despite entering the nucleus with high efficiency, the *TART* Gag protein does not localize to the telomeres on its own and instead requires *HeT-A* Gag [7]. In addition, reverse transcription of the two elements at the end of the chromosome requires the enzymatic activities of the Pol protein. The *TART* Pol protein is composed of two different domains, an endonuclease (EN) and a reverse transcriptase (RT). Because *HeT-A* is a non-autonomous element lacking the *pol* gene, the *TART* Pol protein has been proposed as the most parsimonious solution for obtaining the essential enzymatic activities for *HeT-A* transposition. This potential symbiotic relationship between the two telomeric transposons is conserved across Drosophila species [8,9,10]. The *TAHRE* element combines the presence of a Gag protein, which highly resembles the *HeT-A* Gag protein, and an apparently functional Pol protein [2]. Similarly to *HeT-A* RNA, the *TAHRE* RNA has been observed in the oocyte of different piRNA mutants [11]. Nevertheless, only a few copies of the *TAHRE* element have been found and only in some *D*.*melanogaster* strains [3]. This scenario indicates that *TAHRE* transpositions are occasional and therefore cannot be considered a reliable source for telomere elongation. For this reason we have focused here on the study of the *TART* Pol protein.

Transposable elements (TEs) are potentially deleterious for the genome and several mechanisms of host control have evolved to regulate their transposition [12]. The control of the telomere transposons must have an additional layer of sophistication balancing their selfish nature at the time when the need for telomere elongation is being evaluated. If telomere elongation is needed, transposition of the telomere TEs must be allowed. This balance is especially relevant in germ line tissues where it is necessary to guarantee both the maintenance of telomere length of the cells that will give rise to the next generation and the genomic stability of the progeny [13].

The piRNA (PIWI-interacting RNAs) pathway is a specialized RNAi pathway mainly present in the Drosophila germ line and committed to the control of TEs by cleaving their corresponding RNAs by the principle of complementarity [14]. The existence of different piRNA clusters composed of fragmented and full length TE copies that are transcribed generally in the antisense orientation is also a distinctive feature of control by the piRNA pathway. Interestingly the telomere transposons are not found in the piRNA clusters, although the *HTT* array could be considered as a particular telomeric piRNA cluster. Different PIWI proteins are responsible not only for post-transcriptional gene silencing (PTGS) but also for transcriptional gene silencing (TGS) by interacting with protein complexes involved in the modification of the chromatin. The telomere TEs are under the survey of the piRNA pathway in germ line tissues [14].

Because they are embedded in the telomere chromatin, which itself is specifically regulated, the telomere TEs are also subjected to the action of chromatin regulatory complexes [15]. We have recently described some of the chromatin proteins involved in the epigenetic regulation of Drosophila telomeres [16], [17], [18].

To gain a deeper understanding of how the telomere TEs, *HeT-A* and *TART*, actively contribute to the essential cellular function of telomere maintenance, we have investigated the localization of the different components of the telomere TEs of Drosophila. Our study constitutes the first detailed characterization of the telomere TE proteins and RNAs. Surprisingly, we detect the products of *HeT*-*A* and *TART* in different ovarian cell types at different stages of ovarian development, even though these two retrotransposons are tightly regulated by the piRNA pathway.

## Results

### *HeT-A* and *TART* RNAs are expressed in *wild type* and mutant ovaries

The telomere retrotransposons are expressed at low levels and previous studies have shown that *HeT-A* and *TART* sense RNAs are not detected by *in situ* hybridization in the germ line tissues of the Drosophila ovary in *wild type* conditions [19], [20]. Nevertheless, we have been using the ovary to detect changes in *HeT-A* and *TART* expression through quantitative RT-PCR [21], [22].

To try to visualize the transposition intermediates of the telomere retrotransposons that we have been able to detect by molecular methods, we performed *in situ* hybridization using the Tyramide signal amplification (TSA) method (*see M&M*). We carried out parallel experiments using three different strains: a *wild type* (*wt*) strain; *GIII*, a strain that has longer telomeres and thus higher *HeT-A* and *TART* copy number [23]; and a strain mutant for the piRNA pathway gene *aubergine* (*aub*) in which *HeT-A* and *TART* transcription is de-repressed [24]. Using this sensitive method, we were able to detect expression of both *HeT-A* and *TART* in a *wt* background. The fact that the observed localization of the transcripts was in agreement with previous studies of piRNA pathway mutants [20], [19], together with an increase in the localized signal in the *GIII* and *aub* mutant backgrounds, confirmed the specificity of the probes used in these experiments.

**Fig 1:**
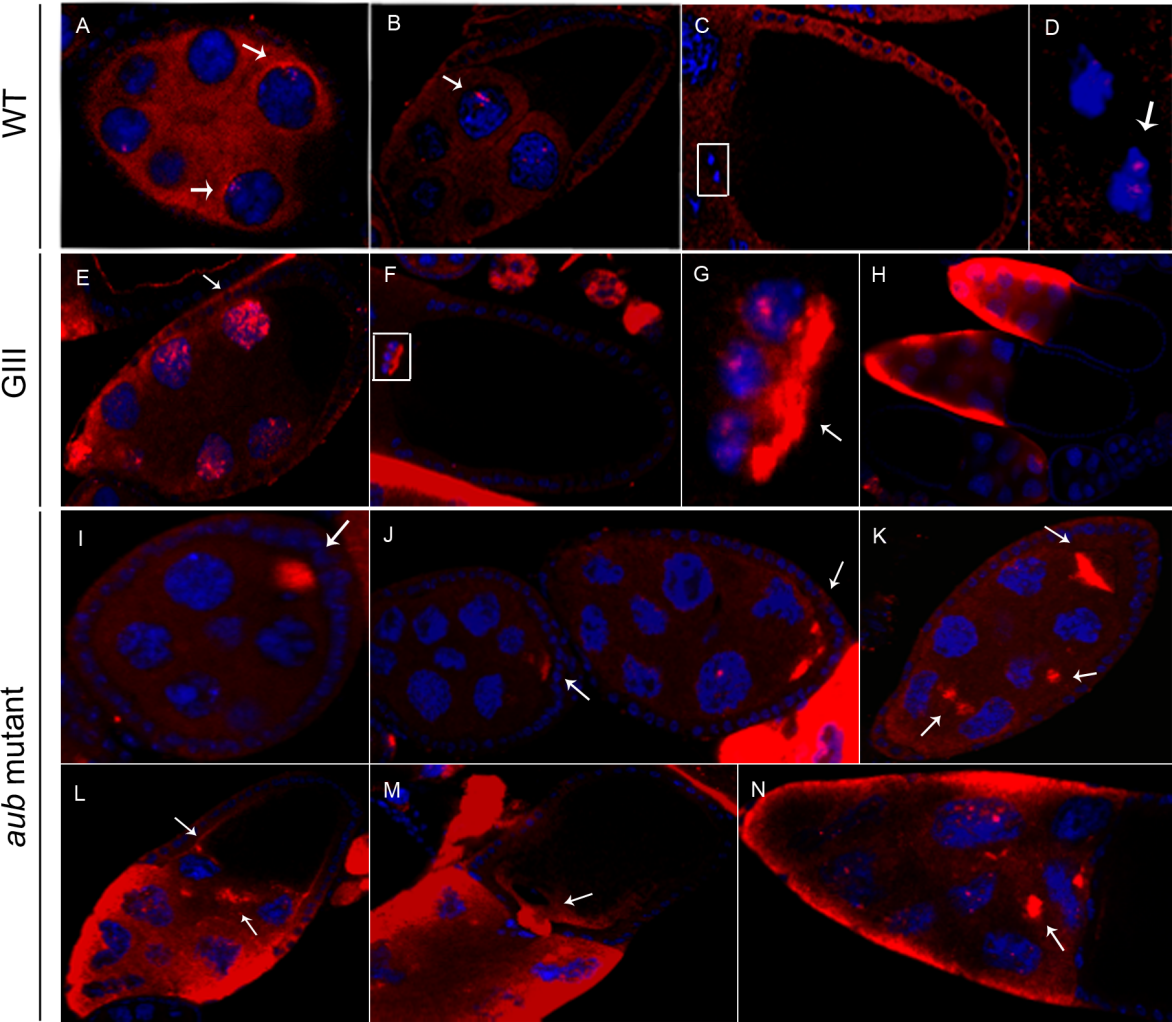
Distribution of *HeT-A* sense RNA in *wt* and mutant ovaries. Fluoresence in situ hybridization to *HeT-A* sense RNA (red). DNA is shown in blue. Images were obtained using 40x magnification, except those specified otherwise. Images for the three genotypes were captured with the same microscope settings. Images in (E-N) have been adjusted to a lower intensity in order to visualize the mRNA distribution. (A-D) *wt* ovaries. (A) Stage 5–6 egg chamber. Arrows indicate localization close to the nucleus of nurse cells. (B) Stage 9. Arrow shows localization inside the nucleus of nurse cells. (C) Stage 10. (D) Magnified view of the border cells boxed in (C). Arrow indicates localization in the nucleus of border cells. (E-H) *GIII* ovaries. (E) Stage 8. Arrow indicates intense localization in the nucleus of nurse cells. (F) Stage 10. (G) Magnification of the border cells boxed in (F). Arrow shows intense localization in the posterior side of border cells cytoplasm. (H) Stages 10a and 10b. This image was obtained with 20x magnification. (I-N) *aub* mutant ovaries. (I) Stage 4. Arrow indicates localization inside the oocyte. (J) Stages 6–7 (left) and 8 (right). Arrows show localization in the oocyte. (K) Stage 8. Arrows indicate localization in the oocyte and in the cytoplasm of nurse cells. (L) Stage 9. Arrows show localization in the oocyte and in the cytoplasm of nurse cells. (M) Stage 10. Arrow indicates localization at the anterior side of the oocyte. (N) Stage 10 nurse cells. Arrow shows detection in the cytoplasm of a nurse cell.

The female germline in Drosophila consists of 12–18 ovarioles each of which starts with the germarium where the stem cells reside and where the oocyte differentiates. Each oocyte is connected at its anterior end to 15 sister germline cells. This 16 cell cluster is surrounded by a somatic follicular epithelium to form an egg chamber [25]. Oogenesis proceeds through 14 morphologically defined stages, ending in the production of a mature oocyte. The *HeT-A* sense is visible in the cytoplasm of both the nurse cells and the somatic follicle cells of *wt* egg chambers beginning at stage 5–6 of oogenesis (Fig 1A–1D). In addition, a few bright dots are observed in some nuclei of the nurse and in the border cells, a specialized set of follicle cells that are important for the formation of the sperm entry structure or micropyle [26] (Fig 1A–1D). Interestingly, these well-defined nuclear dots are also observed with the *TART* Pol protein in the nuclei of the same cells (*see below*).

In a *GIII* background, the distribution of *HeT-A* sense RNA is equivalent to that in *wt* ovaries but in all cases, the RNA levels in the cytoplasm and intranuclear dots of nurse and border cells are dramatically increased (Fig 1E–1H).

In the *aub* mutants there is a slight decrease in the number of bright dots within the nurse cell nuclei (Fig 1I–1N). This result could suggest that *HeT-A* RNA is largely transferred from the nucleus to the cytoplasm. This phenotype would be consistent with a mutant of the piRNA pathway. In addition, the *HeT-A* sense RNA is visible inside the oocyte which likely reflects the increase in RNA levels in the nurse cell cytoplasm (Fig 1I–1M).

Images of *GIII* and *aub* mutant ovaries were captured using the same settings as the ones used for the *wt*. However, because of the substantial increase in *HeT-A* probe signal intensity in the mutants, the images shown in Fig 1E–1N have been adjusted to lower intensity levels in order to reveal the specific RNA distribution.

**Fig 2:**
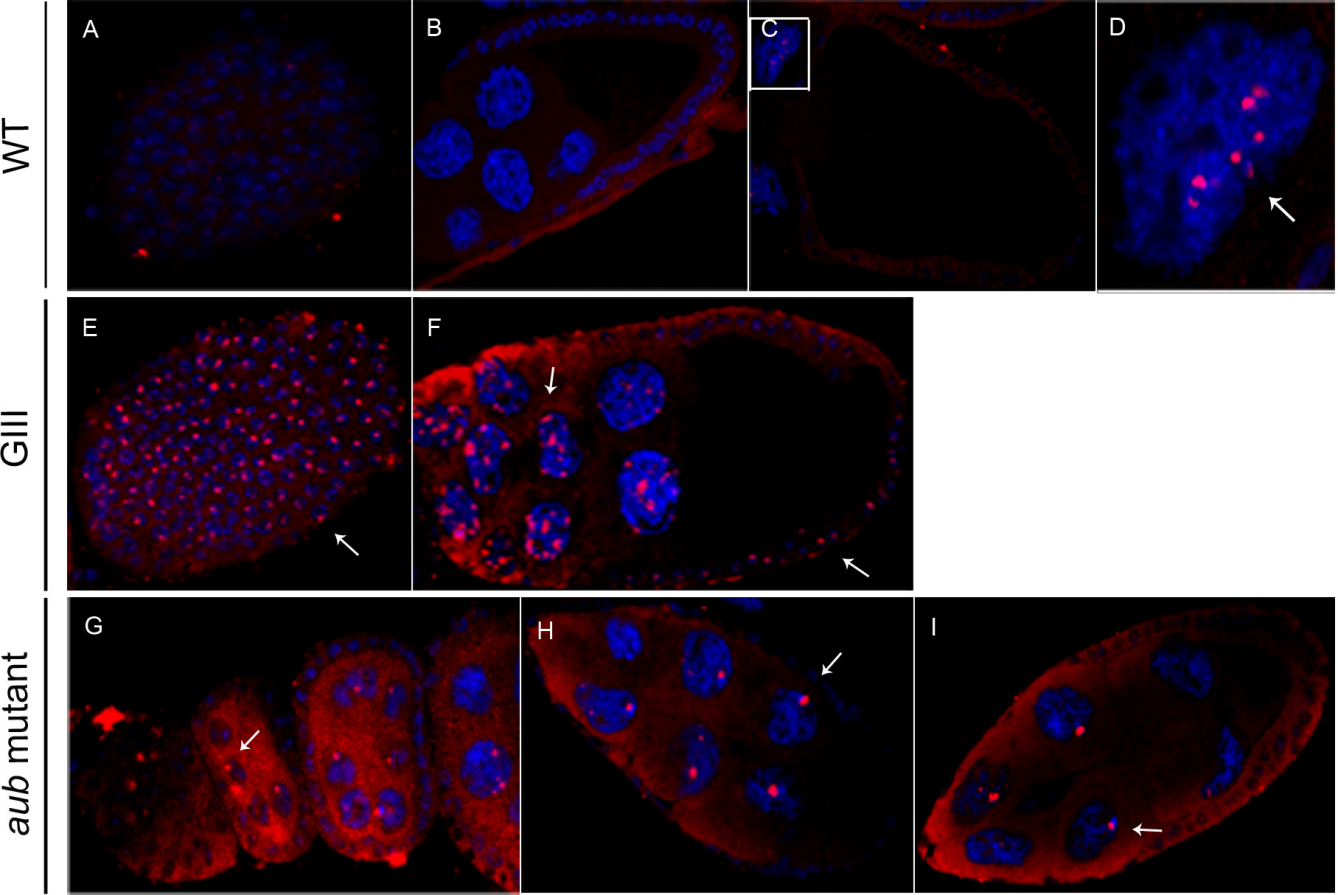
Distribution of the *TART* sense RNA in *wt* and mutant ovaries. Fluoresence in situ hybridization to *HeT-A* sense RNA (red). DNA is shown in blue. Images were obtained using 40x magnification, except for those specified otherwise. (A-D) *wt* ovaries. (A) Follicle cells (external view) of a stage 5–6 egg chamber. (B) Stage 9 egg chamber. (C) Stage 10. (D) Magnification of a nurse cell nucleus from (C). Arrow indicates localization inside the nucleus of a nurse cell. This image was obtained using 63x magnification. (E,F) *GIII* ovaries. (E) Follicle cells (external view) of stage 5–6 egg chamber. Arrow shows localization inside the nucleus of follicle cells. (F) Stage 9. (G-I) *aub* mutant ovaries. Arrows indicate detection in the nucleus of nurse (left) and follicle cells (right). (G) Early stages (germarium and stages 3–4 and 5, from left to right). Arrow shows localization inside the nucleus of a nurse cell. (H) Stage 8. Arrow indicates detection in the nucleus of a nurse cell. (I) Stage 9. Arrow shows localization inside the nucleus of a nurse cell.

When we analyzed the distribution of the *TART* sense RNA in a *wt* background we found that bright dots were observed inside some nurse cell nuclei by the end of stage 10 (Fig 2C and 2D). In the *GIII* background, the same localization was observed but the signal appeared earlier in oogenesis, after stage 5, and faint staining was observed at even earlier stages (Fig 2B and 2F and *data not shown*). In addition, the follicle cell nuclei exhibited a novel, well-defined dot not visible in *wt* (Fig 2A and 2E). In *aub* mutant ovaries, *TART* sense RNA was detected beginning in the germarium and then in the cytoplasm of nurse and follicle cells (Fig 2G and 2H). In contrast to both *wt* and *GIII* ovaries, *aub* mutant nurse cell nuclei contained a single, larger dot (Fig 2B,2F and 2I. In addition, no signal was detected in the follicle cell nuclei (*data not shown*).

Because the telomere retrotransposons have been shown to produce both sense and antisense transcripts [27], [5], we proceeded to analyze the distribution of the antisense RNAs. The level of *HeT-A* antisense RNA was too low to be detected in a *wt* background even with the TSA method (S1A and S1B Fig). In the *GIII* background, the antisense *HeT-A* RNA can be detected in a few nurse cell nuclei as a well-defined dot, but the intensity is always lower than that of the sense *HeT-A* RNA (S1C–S1E Fig). The *TART* antisense RNA has been shown to be more abundant than the sense species in all tissues studied [27], [28], [29], [8], [9]. Consistent with this, we readily observed *TART* antisense RNA in the germ line tissues (S2 Fig). In the *wt* and *GIII* backgrounds the distribution is similar to that of the sense counterpart with one exception: no signal is observed in the follicle cell nuclei (S2 Fig). Interestingly, in *aub* mutant ovaries the *TART* antisense RNA enters the oocyte (S2F–S2I Fig).

### *HeT-A* Gag and *TART* Pol are visible in different ovarian cells and in neuroblasts

In order to better understand the necessary steps of the life cycle of *HeT-A* and *TART*, we investigated the localization of two of the proteins that they encode, *HeT-A* Gag and *TART* Pol, in germ line tissues. We have detected the presence of the telomeric proteins in nurse cells, follicle cells, and border cells. We have also analyzed neuroblasts, the precursors of the larval brain, which are actively diving cells where telomere replication is necessary and therefore provide an opportunity to study the telomere TEs.

**Fig 3:**
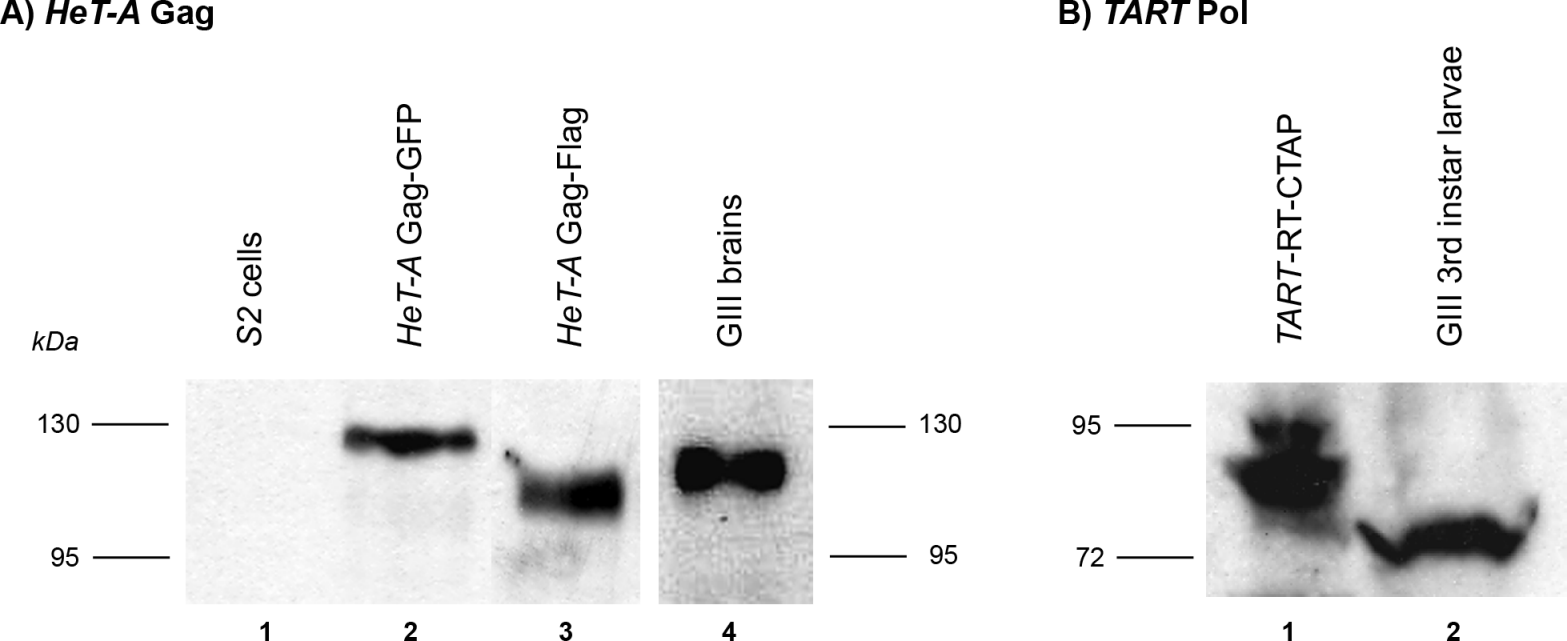
Detection of Gag and Pol proteins in S2 cells and larval brain extracts. (A) Western blot developed with α-*HeT-A* Gag antibody. Lane 1: S2 cells; Lane 2: S2 cells transfected with *HeT-A* Gag-GFP; Lane 3: S2 cells transfected with *HeT-A* Gag-Flag; Lane 4: brains of 3^rd^ instar *GIII* larvae. Both the recombinant and the endogenous HeT-A Gag proteins are detected. (B) Western blot developed with α-*TART* Pol antibody. Lane 1: extract from stable transfected S2 cells *TART*-RT-CTAP; Lane 2: extract from brains of 3^rd^ instar *GIII* larvae. Both the recombinant and the endogenous RT proteins are detected.

To detect these proteins, we generated peptide antibodies that specifically recognize the endogenous *HeT-A* Gag and *TART* Pol proteins. We also generated *HeT-A* and *TART* fusion proteins with GFP, CTAP and Flag tags. We confirmed that the antibody to *HeT-A* Gag recognizes bands of the expected size for *HeT-A* Gag-GFP and Gag-Flag fusion proteins expressed upon transfection of S2 cells as well as the endogenous protein in brains of 3^rd^ instar *GIII* larvae (Fig 3A). In the case of *TART* Pol, the antibody was generated using a peptide sequence from the RT domain. We generated stably transfected cells with the *TART* RT domain fused to the CTAP tag. The anti-*TART* Pol antibody recognizes a band of the expected size for the *TART* RT-CTAP protein in S2 cells as well as the endogenous protein in brains of 3^rd^ instar *GIII* larvae (Fig 3B). The specificity of these antibodies in immunolocalization experiments was verified by comparison of *wt*, *GIII*, and *aub* mutant tissues (see above).

**Fig 4:**
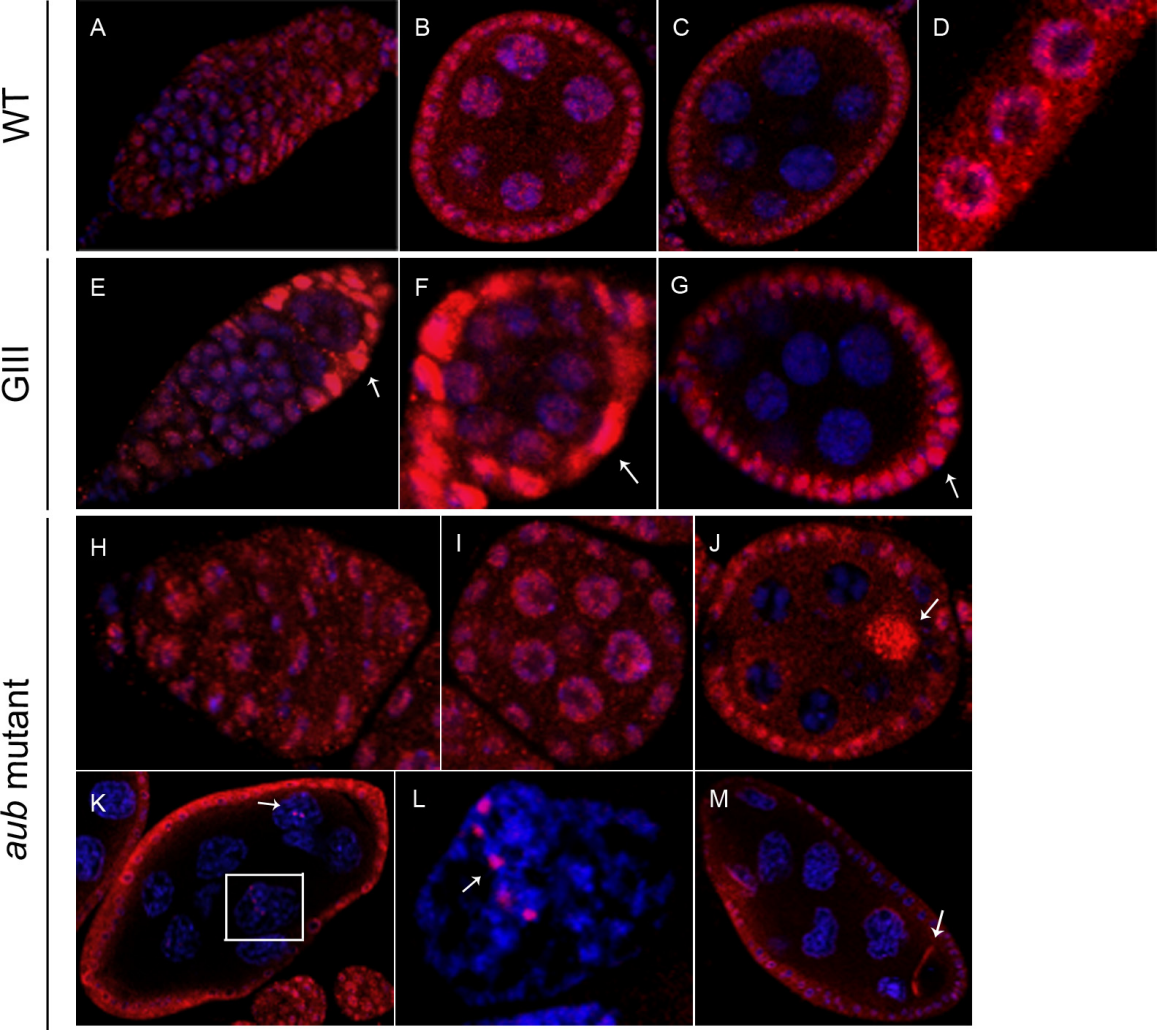
Localization of *HeT-A* Gag protein in *wt* and mutant ovaries. Immunofluorescence detection of *HeT-A* Gag (red). DNA is stained in blue. Images were obtained using 40x magnification, except those specified otherwise. Arrowheads correspond to differences in localization between mutant and *wt* ovaries. (A-C) *wt* ovaries. (A) Germarium and stage 1 egg chamber. (B) Stage 3–4. (C) Stage 5–6. (D) Magnified image of follicle cells. (E-G) *GIII* ovaries. (E) Germarium and stage 1. Arrow indicates high levels of detection in follicle cells. (F) Stage 2–3. Arrow shows high levels of detection in follicle cells. (G) Stage 5–6. Arrow indicates intense signal in follicle cells. (H-M) *aub* mutant ovaries. (H) Germarium (I) Stage 2–3. (J) Stage 4–5. Arrow shows localization in the oocyte. (K) Stage 8. Image obtained using 20x magnification. Arrow indicates detection in the nucleus of nurse cells. (L) Magnification of boxed nurse cell nucleus in (J). Image obtained using 63x magnification. Arrow shows localization in the nucleus of a nurse cell. (M) Stage 8. Image obtained using 20x magnification. Arrow indicates detection in the oocyte.

Our results indicate that in *wt*, *HeT-A* Gag is found at the first stages of oogenesis in both, nurse and follicle cells. In nurse cells, *HeT-A* Gag is especially abundant in the nucleus and also clearly visible in the cytoplasm but gradually disappears and is no longer present by stage 5–6 (Fig 4A–4C). In the follicle cells, *HeT-A* Gag is found mainly in the cytoplasm around the nuclear membrane, where it persists through subsequent stages (Fig 4B–4D). When *HeT-A* Gag is analyzed in a *GIII* background, the main difference is observed in the follicle cells, where *HeT-A* Gag is significatively increased from the beginning until stage 6 of oogenesis (Fig 4E–4G). In *aub* mutants, expression of *HeT-A* Gag in the nurse cells and follicle cells persists to at least stage 8 (Fig 4H–4M). Most notably, *HeT-A* Gag accumulates in the developing oocyte (Fig 4J and 4M) and also forms well-defined bright dots within the nurse cell nuclei (Fig 4K and 4L).

**Fig 5:**
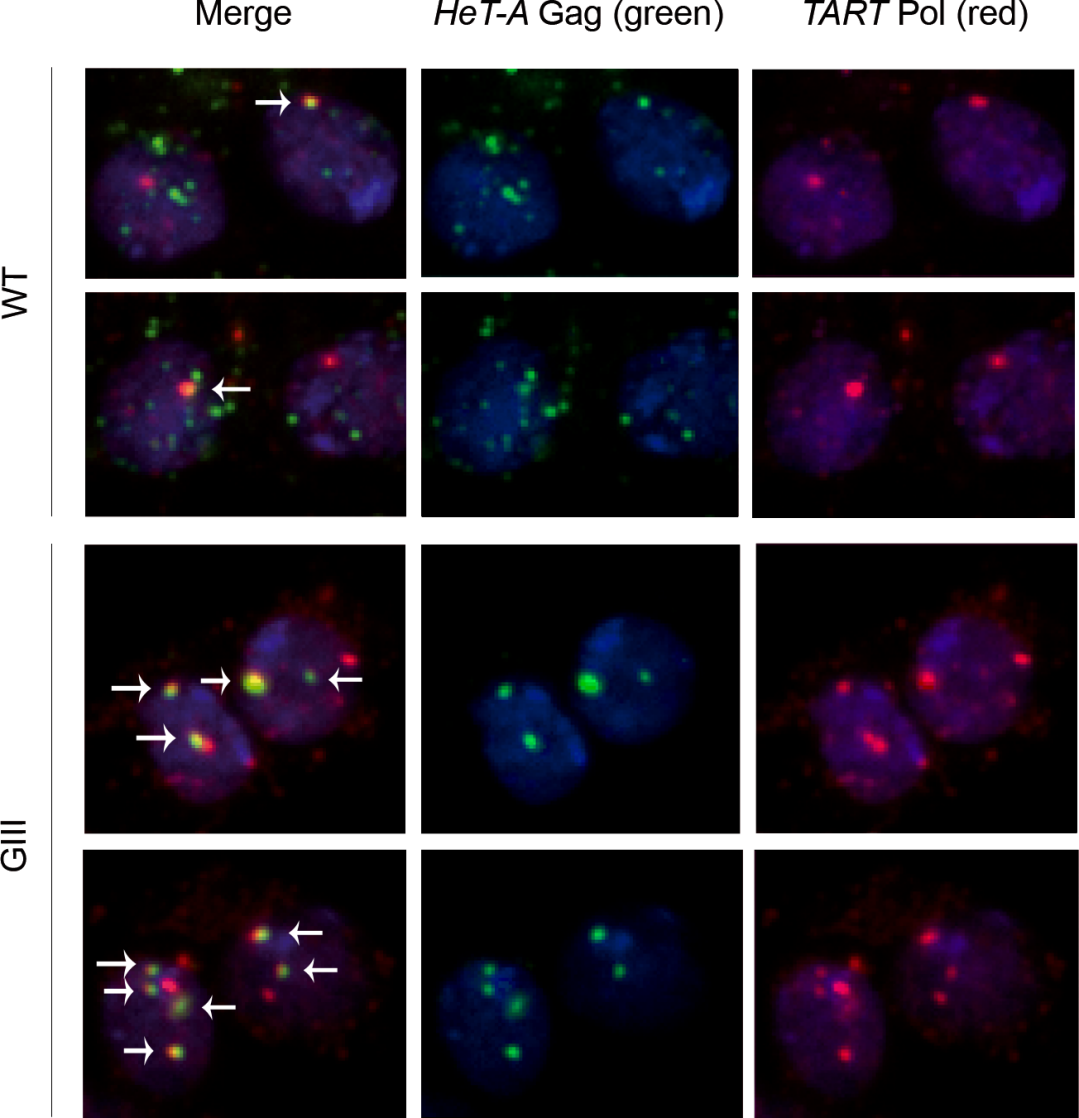
Localization of the *HeT-A* Gag and *TART* Pol proteins in *wt* and mutant neuroblasts. Immunofluorescence detection of *HeT-A* Gag (green) and *TART* Pol (red). DNA stained in blue. First column (at left): merge of the three channels. Second column (middle): *HeT-A* Gag (green). Third column (at right): *TART* Pol (red). First and second row: *wt* neuroblasts. Third and fourth row: *GIII* neuroblasts. Arrowheads indicate co-localization of *HeT-A* Gag and *TART* Pol (visible in yellow).

We have detected both *HeT-A* Gag and *TART* Pol proteins in *wt* neuroblasts (Fig 5). *HeT-A* Gag and *TART* Pol co-localize in several spots that correspond to telomere clusters near the nuclear membrane [30], [31]. In the case of neuroblast preparations from the *GIII* strain, similar nuclear dots were observed but this time with an increased level of co-localization of both proteins (Fig 5).

**Fig 6:**
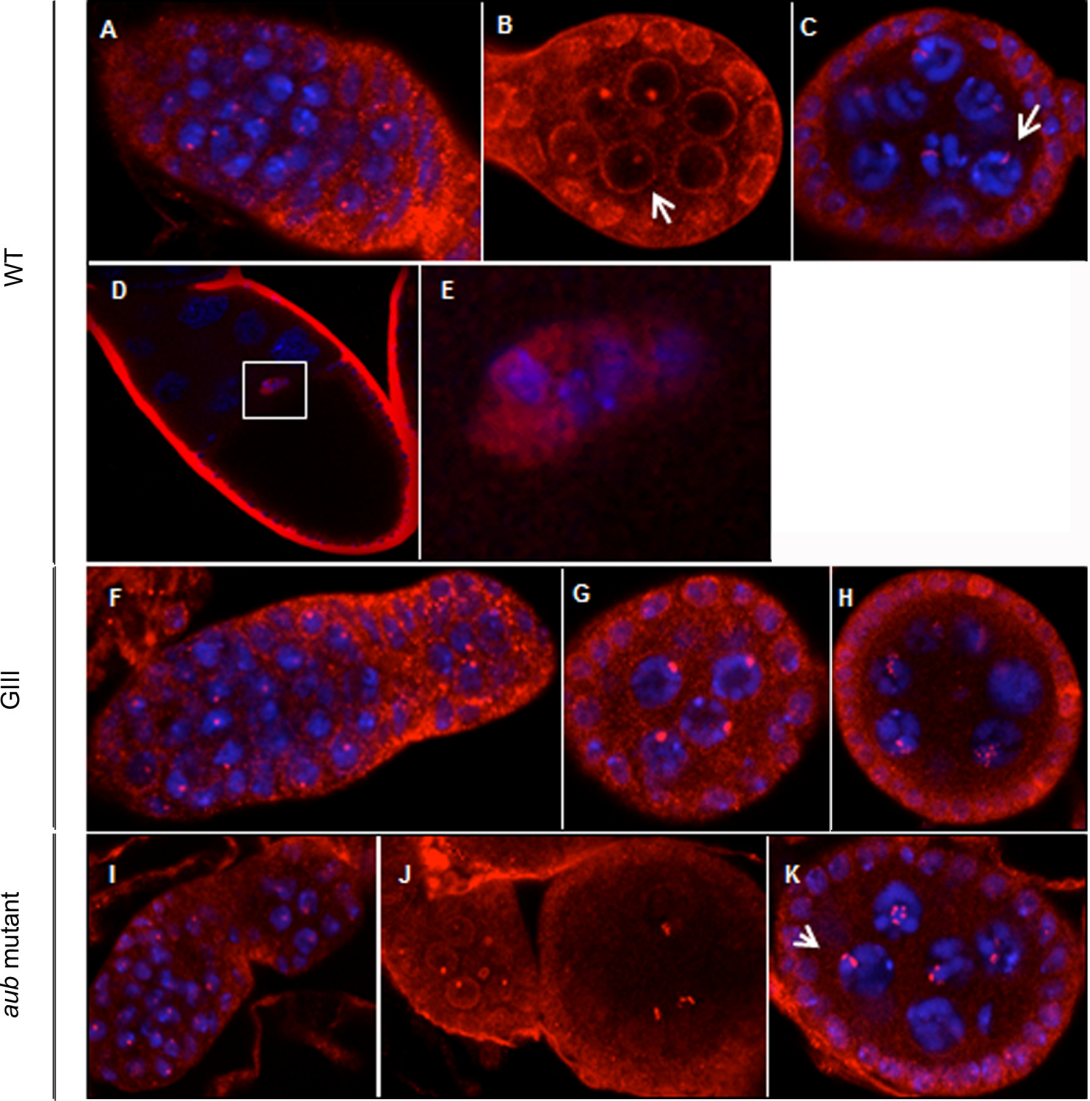
Localization of the *TART* Pol protein in *Drosophila melanogaster wt* and mutant ovaries. Immunofluorescence detection of *TART* Pol (red). DNA stained in blue except for (B). Images were obtained using 40x magnification, except those specified otherwise. *(*A-E) *wt* ovaries (A) Germarium. (B) Stage 2–3. Arrow shows perinuclear detection in nurse cells. (C) Stage 4. Arrow indicates localization inside the nucleus of nurse cells. (D) Stage 10. Image obtained with 20x magnification. (E) Magnified view of the border cells in (D) (posterior up). (F-H) *GIII* ovaries. (F) Germarium and stage 1. (G) Stage 2–3. (H) Stage 4–5. (I-K) *aub* mutant ovaries. (I) Germarium and stage 1–2. (J) Stages 2 and 4. (K) Stage 5–6. Arrow indicates localization in the nucleus of nurse cells.

*TART* Pol shows a distribution similar to *HeT-A* Gag (Fig 4D) in the follicle cells, accumulating close to the nuclear membrane-(Fig 6B). *TART* Pol also accumulates around the nurse cell nuclei during the first stages of oogenesis but this localization disappears by stage 4 (Fig 6A–6C). In addition, a single bright dot of *TART* Pol is observed within each nurse cell nucleus at stages 2–3 (Fig 6B). By stage 4, each nucleus contains several dots that often appear as strings (Fig 6C). In both *GIII* and *aub* mutant backgrounds, the distribution of *TART* Pol is very similar to that in *wt*, but the protein persists to later stages and there is an increased number of intranuclear dots at stages 4–5 (Fig 6F–6K). In addition, *TART* Pol is clearly visible in both the nucleus and cytoplasm of the border cells from the beginning of their migration (stage 9) (Fig 6D and 6E). This unexpected distribution is shared with another component of the telomeric RNP, the *HeT-A* RNA. The levels of both *TART* Pol and *HeT-A* sense RNA in the cytoplasm and nucleus of border cells are significatively increased in a *GIII* background (Fig 1F and 1G) (*see previous section and Discussion*).

In summary, we have found both *HeT-A* Gag and *TART* Pol in the nuclei of the nurse cells as well as in the cytoplasm of the follicle cells until stage 6 of oogenesis. Interestingly, this is the point when follicle cells stop undergoing mitosis and begin endoreplication cycles [32]. *TART* Pol is also observed at the border cells.

### The well-defined dots of *TAR*T Pol in nurse cells are not the telomeres

**Fig 7:**
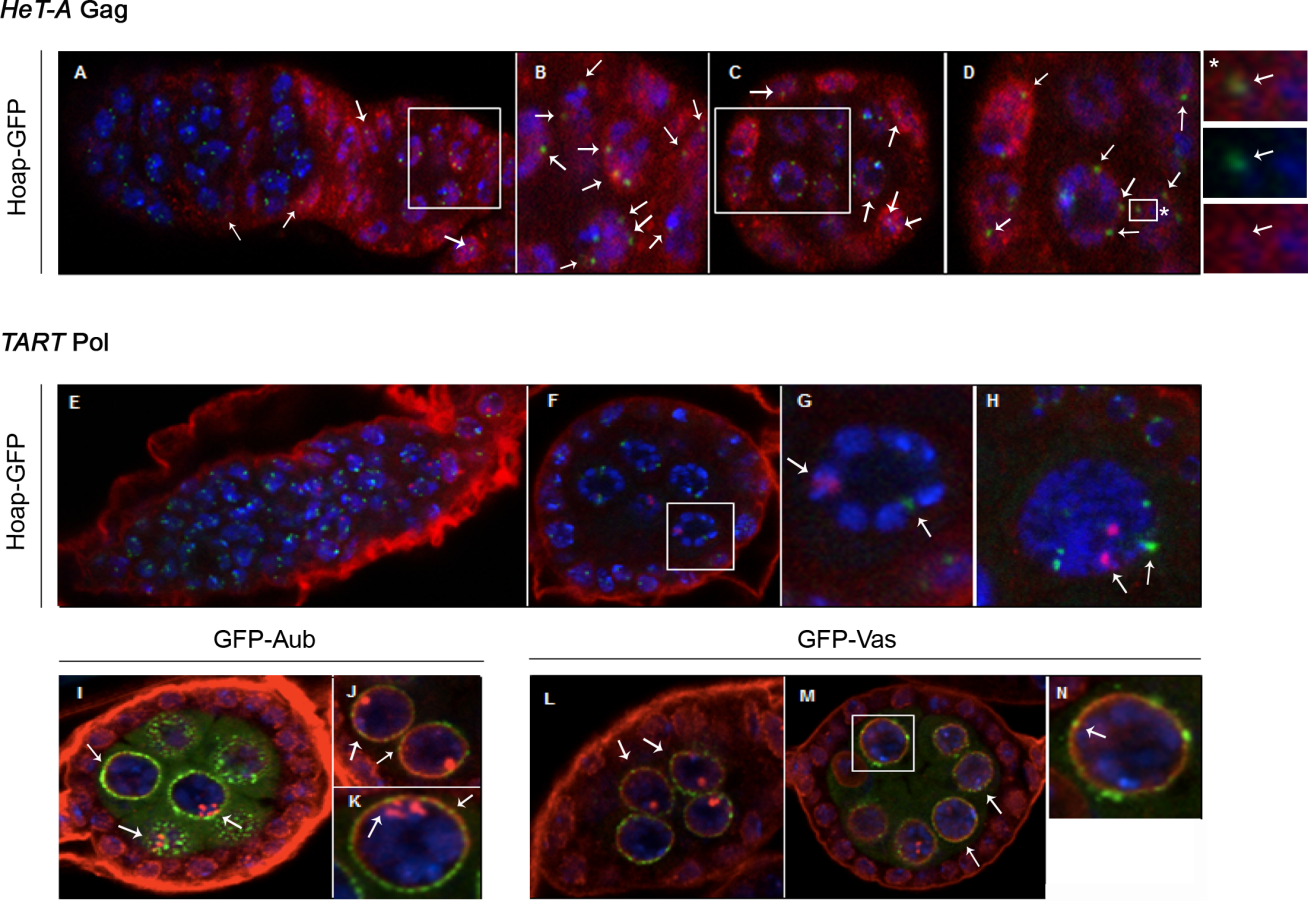
Co-localization of telomere proteins *HeT-A* Gag or *TART* Pol with Hoap and *nuage* proteins. (A-D) Immunofluorescence detection of *HeT-A* Gag protein (red) in Hoap-GFP (green) ovaries. DNA stained in blue. Arrows show the co-localization of the two proteins (yellow). (A) Germarium and stage 1–2 (from left to right). (B) Magnified view of boxed region in (A). (C) Stage 3–4. (D) Magnification of boxed region in (C). (*) Magnification of boxed region in (D): panel on top shows merged colors; panel in the middle shows Hoap localization (green); panel below shows *HeT-A* Gag localization (red). (E-N) Immunofluorescence detection of *TART* Pol protein (red) in Hoap-GFP (green), Aubergine-GFP (green) and Vasa-GFP (green) ovaries. DNA stained in blue. (E) Hoap-GFP germarium and stage 1. (F) Hoap-GFP stage 3–4. (G) Magnification of a nurse cell nucleus from (F). (H) Magnified view of Hoap-GFP stage 4 nurse cell nucleus. Arrows show the co-localization of the two proteins (yellow). (I) GFP-Aub stage 3–4. (J, K) GFP-Aub stage 3–4, magnified view of nurse cell nuclei. (L) GFP-Vasa stage 2. (M) GFP-Vasa stage 3–4. (N) Magnification of nurse cell nucleus boxed in (M).

We investigated if the well-defined bright dots within the nurse cell nuclei that contain both *HeT-A* Gag and *TART* Pol correspond to the telomeres. Thus, we detected both proteins in egg chambers expressing the telomere protein Hoap (Heterochromatin Protein 1 and ORC associated protein) fused to GFP to mark the telomeres [33]. The co-localization of *HeT-A* Gag with Hoap confirmed its telomeric localization (Fig 7A–7D). In contrast, *TART* Pol and Hoap do not co-localize, indicating that *TART* Pol is not on the telomeres (Fig 7E–7H).

### *TART* Pol is found at the *nuage* structure

The peri-nuclear localization of the *TART* Pol protein in the nurse cells is reminiscent of the *nuage* structure, a specialized structure essential for the processing of the piRNAs [14]. Therefore we proceeded to investigate if *TART* Pol has the same localization as known components of the *nuage* structure such as Aub and Vasa (Vas). We co-visualized *TART* Pol together with GFP-Aub (Fig 7I–7K) or Vas-GFP (Fig 7L–7N). Only a few regions of co-localization between *TART* Pol and Aub (Fig 7I–7K) or Vas (Fig 7L–7N) were observed, suggesting that *TART* Pol is only partially found in the *nuage*.

## Discussion

### The RNAs of the telomere TEs are present at different stages of the germ line development

As previously demonstrated, *HeT-A* and to a lesser extend *TART* are targets of the piRNA machinery [34], [20], [19]. Transposition in germ line tissues is dangerous because it results in new insertions that will be inherited by the next generation and therefore, control of transposable elements in these tissues is especially important [14], [11]. By comparing a *GIII* background, which has increased expression of the telomeric transposons but maintains the mechanisms of TE control as in *wt*, with *aub* mutants that are impaired in piRNA pathway function, we have provided additional evidence for such control over *HeT-A* and *TART*. The observed decrease in the amount of *HeT-A* and *TART* RNA within the nurse cell nuclei in *aub* mutants is consistent with increased export of the transposon mRNAs from the nucleus to the cytoplasm. Accordingly, the bright dots observed in the *wt* and *GIII* backgrounds could be explained as accumulation of the sequestered *HeT-A* and *TART* RNAs inside the nuclei resulting from the action of the piRNA control at this step of their life cycle and confirming once more the survey of the telomere RNAs by the piRNA machinery in both the *wt* and *GIII* backgrounds. Similar results have been obtained for other non-LTR retrotransposons such as the *I* factor [35]. In accordance with an increased transfer of *HeT-A* RNA to the cytoplasm, *HeT-A* Gag protein is also detected in the nurse cells of this mutant background.

In spite of the high efficiency of the piRNA mechanism, TEs occasionally manage to escape this control and new transpositions are sometimes possible [36]. When this occurs, new transpositions are not necessarily deleterious and become the substrate for variability, thereby increasing the plasticity of the genome. The possible benefit of *HeT-A* and *TART* transposition goes beyond genome plasticity since their transpositions are needed to maintain the receding end of the telomeres after each cell division [36], [37]. Therefore, the established control over the telomere retrotransposons must accommodate exceptions depending on the need for telomere replication at each stage. How *HeT-A* and *TART* manage to escape the control by the piRNA pathway is not yet known, suggesting that additional mechanisms by which TEs manage to escape genome control remain to be discovered.

### *HeT-A* Gag and *TART* Pol co-localize at the telomeres in telomere replicating cells such as the neuroblasts

It was believed that telomere replication was only performed when terminal erosion drove telomere length to a critical limit. Studies in vertebrates as well as in Drosophila indicate that telomeres are very dynamic, being extensively reset and built *de novo* at certain steps of development [38]. In this scenario, several sequential transpositions would be needed in order to replenish the receding telomere length in a short period of time. Accordingly, we and others have demonstrated that several telomeres of different *D*.*melanogaster* strains have an *HTT* structure and composition in agreement with this hypothesis [21] [39].

The larval neuroblast populations are the precursors of the adult neurons. These neuroblasts divide hundreds of times [40], beginning in the first larval instar stage and continuing until early pupal development. Different studies have demonstrated that the larval brain is one of the developing tissues with higher expression of the telomere retrotransposons [41], [16] and, more importantly, our previous work demonstrated that this high rate of mitotic divisions requires telomere replenishing since impairment of this process is highly deleterious [16]. We have now shown that both proteins, *HeT-A* Gag and *TART* Pol accumulate in foci in the nuclei of these cells. Co-localization was observed in several spots close to the nuclear membrane, which have previously been identified as telomeres [30], [31]. Neuroblast preparations from the *GIII* strain show the same pattern of foci. The co-localization of *HeT-A* Gag and *TART* Pol in these highly dividing cells together with their simultaneous presence at the follicle cells until stage 6, the stage at which they stop proliferating and enter the endocycles, suggests that *HeT-A* Gag and *TART* Pol co-localize when the cell is undergoing division in addition of replication.

Both, nurse and follicle cells undergo several rounds of endoreplication in order to provide the oocyte with the required components as well as the eggshell. Interestingly, we have found no evidence for the presence of the telomere RNP (proteins or RNAs) in endoreplicative tissues such as salivary glands or the adult tracheal progenitors (*data not shown*). Thus it was surprising to find *HeT-A* Gag and *TART* Pol in the nuclei of endoreplicating nurse cells and follicle cells. Their accumulation may be related to an alternative function other than telomere replenishing which does not require their co-localization. Recently, extra-telomeric roles have been described for several telomere components in other organisms, like fission and budding yeast as well as mammals [42]. For example, telomerase-spliced forms have been described that are important to stimulate proliferative capacity without telomere elongation. Interestingly, some of these spliced forms seem to be conserved in evolution [43].

### *TART* Pol and *HeT-A* RNA co-localize in the border cells

Border cells are a subset of follicle cells that are recruited by the anterior polar follicle cells at stage 8 to encircle them and form a migratory cell cluster [44]. Neighboring polar cells start to signal through the production of Unpaired (UPD), which activates the Janus Kinase (JAK), whose phosphorylation activity will activate the Signal Transducer and Activator of Transcription (STAT). STAT then activates a downstream pathway. Ken, a DNA binding protein is among the different downstream targets of the JAK-STAT pathway [45]. Interestingly, we have recently demonstrated that Ken is involved in the control of Drosophila telomeres by regulating the expression of the *TART* retrotransposon through specific binding sites [17].

*HeT-A* is the most active of the three telomere retrotransposons and *TART* Pol is the best candidate to supply the enzymatic activities for *HeT-A* transposition [3]. The presence of *TART* Pol and *HeT-A* RNA from stage 9 onwards in the border cells, when the polar cells activate the signaling pathway responsible for activating Ken, suggests the possibility that *HeT-A* might be actively transposing in these cells. Although involvement of the telomere retrotransposons in the fate of the border cells seems unlikely, our results suggest a connection between the biology of these cells, telomere replication and/or TEs life cycle.

### Unexpected localization of the telomere TE proteins

Interestingly, the location of bright foci of *TART* Pol over regions of nurse cell nuclei distant from the telomeres coincides with the localization of another component of the telomere RNP, the *HeT-A* RNA. As extra-telomeric roles for different telomere components in yeast and mammals have been recently demonstrated [42], it is possible that the non-telomeric localization of *TART* Pol and the *HeT-A* RNA reflects an alternative function of these components. Telomere components like some of the shelterin units have been shown to bind and act as transcription factors for many non-telomeric genes [46], [47]. Moreover, as mentioned earlier, alternative spliced forms of telomerase are able to induce cell proliferation without inducing changes in telomere biology [42]. Finally, the presence of both *HeT-A* Gag and *TART* Pol proteins and the *HeT-A* RNA in the nurse cell nuclei opens the possibility that the *HeT-A* elements might be opportunistically transposing inside these cells although the new transpositions in nurse cells will not be passed on the next generation.

## Conclusions

Our study shows for the first time the localization of the different components of the telomere RNP in Drosophila. *HeT-A* Gag and *TART* Pol, two of the main components of the telomere RNP, co-localize in telomere replicating cells as shown for neuroblasts. In addition, the presence of *TART* Pol and the *HeT-A* sense RNA in the border cells points to an alternative and extra-telomeric role for these telomere components in this cellular type.

Finally, subtle changes in telomeres or extra-telomeric effects of telomere components have been suggested to be pivotal for different cell types or tissues to adapt to their specific environment or developmental stage [42]. Essential processes such as mitochondrial function, energy metabolism or time of replication are controlled by telomeric proteins in mammals and yeast, respectively [42]. Therefore, the description of the localization of the Drosophila telomere proteins in different tissues provides insight on how telomere biology and the telomere retrotransposons in Drosophila might be linked to several essential cellular processes.

## Materials and Methods

### Generation of primary antibodies

Antibodies were generated from two immunized rabbits, after inoculation of the synthesized peptide. Peptide for anti-*HeT-A* Gag antibody: AAIKIVNSLSHKKKEC. The peptide for anti-*TART* Pol antibody is from the Reverse transcriptase domain (RT): FSETIKSHPNIAVRRC.

Rabbit immunizations included in this study were carried out in the animal facility of the Research and Development Center (CID) from the Spanish Research Council (CSIC)-Registration Number: B9900083-, in strict accordance with the bioethical principles established by the Spanish legislation, which follows the international agreements on that matter. All efforts were made to minimize suffering of the animals. The protocol used for the production of antibodies was approved by the Committee of Bioethics and Animal Experimentation of CID and notified to the competent authorities.

### Fly stocks

Fly stocks were maintained at 25°C on standard Drosophila corn meal medium. The w^1118^ strain was used as the *wt* control. The *Gaiano III* (*GIII*) strain carries the third chromosome from the Gaiano strain (with the *Tel-1* mutation) in an Oregon-R background. The *aub* ^*QC42*^ and *aub* ^*HN2*^ alleles (Schüpbach and Wieschaus, 1991) were used in trans-heterozygous combination to generate *aub* mutants animals. Transgenic stocks expressing *GFP-Aub* (Snee and Macdonald, 2004) and *GFP-Vas* (Johnstone and Lasko, 2004) have been previously described. The stock Hoap-GFP HipHop^mCherry^ was generated and provided by Dr. Yikang Rong (NIH, USA).

### Immunofluoresence (ovaries)

Ovaries from young females fed for 2–3 days on yeast were dissected in PBS and fixed in 4% EM-grade formaldehyde (Polysciences) for 15 min. Washes were performed with PBST (PBS/0.1% Tween-20). Fixed ovaries were treated with Image-iT FX Signal Enhancer (Molecular Probes-Invitrogen) in the dark for 30 min and blocked for 1 hr with BBT [PBST/0.1% globulin-free BSA (Bovine Serum Albumin)]. Rabbit α-*HeT-A* Gag or Rabbit α-*TART* Pol Primary antibodies were diluted 1:50 in BBT and incubated overnight at 4°C. Ovaries were washed, blocked in BBT + 2% NGS (Normal Goat Serum) for 1 hr, and then incubated with Alexa Fluor 555 goat anti-rabbit secondary antibody (Invitrogen) diluted 1:500 in BBT + 2% NGS for 1 hr. DNA was stained with To-Pro-3 (1:1000) for 30 min. Samples were mounted in Vectashield (Vector laboratories) and imaged with a Leica SPE confocal microscope using 20x, 40x and 63x objectives.

### Immunofluoresence (neuroblasts)

Brains of 3^rd^ instar larvae were dissected in 0.7% NaCl, incubated for 10 min in 0.5% Trisodium citrate and fixed 8 min in 3.7% formaldehide + 45% acetic acid on a cover-slip pre-treated with Silanization Solution II (Fluka). Brains were squashed and washed in 0.1% PBT (PBS/0.1% Triton X-100) and blocked in PBTM (PBT/1% non-fat dry milk) for 1 hr. Samples were incubated with α-*HeT-A* Gag (guinea pig; obtained from Dr. Yikang Rong) 1:1000 and α-*TART* Pol (rabbit) 1:50 primary antibodies diluted in PBTM overnight in a humid chamber at 4°C. A wash with PBT was followed by 3, 15 min washes with PBTM. Slides were incubated with secondary antibodies [Alexa Fluor 488 goat anti-guinea pig (Invitrogen), Alexa Fluor 555 goat anti-rabbit (Invitrogen)] in PBTM 1 hr at room temperature in the dark and washed with PBT and PBS. A drop of DAPI-containing Mowiol medium was added to the slide and covered with a coverslip. Images were obtained using the Zeiss Axio Imager.Z2 fluorescence microscope using 20x, 40x and 63x objectives.

### Fluorescence *in situ* hybridization of ovaries

Ovaries were dissected, fixed, and washed as described for immunofluorescence. In situ hybridization was performed as described in [26]. Tyramide signal amplification was performed for 30 min with Alexa Fluor 568 Tyramide (TSA kit; Invitrogen-Molecular Probes) according to the manufacturer's instructions. DNA was stained with To-Pro-3 (1:1000) for 30 min. Samples were mounted in Vectashield (Vector laboratories), and images were obtained using a Leica SPE confocal microscope using 20x, 40x and 63x objectives.

Probes used for the in situ hybridization

Full-length sequences of *HeT-A* Gag and *TART* Pol were digested with PstI/HindIII and PstI/SacI respectively and re-cloned into the pSTBlue-1 vector in order to synthesize sense and antisense probes by *in vitro* transcription using the Sp6/T7 DIG RNA Labeling Kit (Boehringer).

### Transient transfection of S2 cells

*Drosophila* S2 cells were seeded at 3 x 10^6^ cells/ml and grown in Schneiderʼs medium (Sigma) in 10% fetal bovine serum at 25°C. Cells were transfected with 1 μg of the recombinant construct containing the desired fusion protein (*HeT-A* Gag-GFP and *HeT-A* Gag-Flag). The *Effectene* transfection reagent (Qiagen) was used according to manufacturer’s instructions. At 48 hr after transfection, cells were washed once in PBS, and were prepared for microscope observation or pelleted and frozen at -80°C for protein immunoprecipitation.

### Generation of stable transfected S2 cell lines

The *TART*-RT domain was amplified in order to eliminate the natural stop codon and was subcloned into the pMK33-CTAP vector [48] using a *BamHI* restriction.

*Drosophila S2* cells were grown in Schneiderʼs medium (Sigma) in 10% fetal calf serum at 25°C. Cells were transfected with 2 μg of pMK33-based construct (*TART*-RT-CTAP) using Effectene transfection reagent (Qiagen). After three days of incubation, cells were subjected to selection in the presence of 300 μg/ml of Hygromycin B (Sigma). Cell medium was changed every week without disturbing the selected cells. Stable cell lines were established after approximately one month.

Optimization of the necessary amount of CuSO_4_ to induce production of the desired fusion protein from the stable established cell line was performed. *TART*-RT-CTAP cells were induced with 200 μM CuSO_4_ for 24 hours. Then, cells were collected, centrifuged 5 min at 1800 rpm, washed with PBS, and frozen at -80°C.

### Protein immunoprecipitation of S2 cells and larvae brains

Protein extracts from S2 cells or brains of 3^rd^ instar larvae were prepared in 1 ml lysis buffer *SB* (50 mM Tris-HCl pH 7.4, 100 mM NaCl, 1% TritonX-100, 1 mM EDTA, 1 mM EGTA, and Complete EDTA-free protease inhibitor cocktail from Roche), incubated on ice for 20 min, and centrifuged at 13000 rpm for 10 min at 4°C. Fresh lysates were incubated with 50 μl PureProteome Protein A and Protein G Magnetic Beads (Millipore) coated with specific antibodies, for 2 hours at 4°C with rotation. The magnetic beads were previously incubated with the respective antibodies in 1 ml lysis buffer for 1 hour at 4°C with rotation and washed 3 times with 1 ml lysis buffer. Immunocomplexes were washed 6 times with lysis buffer and eluted from the beads with 40 μl sample buffer. Samples were boiled for 10 min, loaded on an SDS-PAGE gel and analyzed by Western Blot. Mouse α-GFP antibody (Invitrogen, A11120) was used for protein immunoprecipitation, and rabbit α-TAP (Thermo Scientific) and mouse α-GFP (Roche) antibodies were used in Western Blot experiments.

## Supporting Information

S1 Fig Antisense *HeT-A* RNA in germ line tissues. (TIFF)

S2 Fig Antisense *TART* RNA in germ line tissues. (TIFF)

## References

[1] Sahara K, Marec F, Traut W (1999) TTAGG telomeric repeats in chromosomes of some insects and other arthropods. Chromosom Res 7: 449–460. 10560968 10.1023/a:1009297729547

[2] Villasante A, Abad JP, Planelló R, Méndez-Lago M, Celniker SE, de Pablos B (2007) Drosophila telomeric retrotransposons derived from an ancestral element that was recruited to replace telomerase. Genome Res 17: 1909–1918. 10.1101/gr.6365107 17989257 PMC2099598

[3] Silva-Sousa R, López-Panadès E, Casacuberta E (2010) Drosophila telomeres: an example of co-evolution with transposable elements. Genome Dyn 7: 46–67. 10.1159/000337127 22759813

[4] Pardue M-LL, DeBaryshe PG (2008) Drosophila telomeres: A variation on the telomerase theme. Fly 2: 101–110. 18820466 10.4161/fly.6393

[5] Casacuberta E, Pardue M-L (2005) HeT-A and TART, two Drosophila retrotransposons with a bona fide role in chromosome structure for more than 60 million years. Cytogenet Genome Res 110: 152–159. 10.1159/000084947 16093667 PMC1188233

[6] Rashkova S, Karam SE, Kellum R, Pardue M-L (2002) Gag proteins of the two Drosophila telomeric retrotransposons are targeted to chromosome ends. J Cell Biol 159: 397–402. 10.1083/jcb.200205039 12417578 PMC2173066

[7] Rashkova S, Athanasiadis A, Pardue M-L (2003) Intracellular targeting of Gag proteins of the Drosophila telomeric retrotransposons. J Virol 77: 6376–6384. 12743295 10.1128/JVI.77.11.6376-6384.2003 PMC155015

[8] Casacuberta E, Pardue M-L (2003) HeT-A elements in Drosophila virilis: retrotransposon telomeres are conserved across the Drosophila genus. Proc Natl Acad Sci U S A 100: 14091–14096. 10.1073/pnas.1936193100 14614149 PMC283551

[9] Casacuberta E, Pardue M-L (2003) Transposon telomeres are widely distributed in the Drosophila genus: TART elements in the virilis group. Proc Natl Acad Sci U S A 100: 3363–3368. 10.1073/pnas.0230353100 12626755 PMC152298

[10] Casacuberta E, Mar’in FA, Pardue M-L (2007) Intracellular targeting of telomeric retrotransposon Gag proteins of distantly related Drosophila species. Proc Natl Acad Sci U S A 104: 8391–8396. 10.1073/pnas.0702566104 17483480 PMC1895960

[11] Shpiz S, Olovnikov I, Sergeeva A, Lavrov S, Abramov Y, Savitsky M, et al (2011) Mechanism of the piRNA-mediated silencing of Drosophila telomeric retrotransposons. Nucl. Acids Res. 39: 8703–8711 doi: 10.1093 21764773 10.1093/nar/gkr552 PMC3203600

[12] González J, Petrov DA (2012) Evolution of genome content: population dynamics of transposable elements in flies and humans. Methods Mol Biol 855: 361–383. 10.1007/978-1-61779-582-4_13 22407716

[13] Shpiz S, Kalmykova A (2011) Role of piRNAs in the Drosophila telomere homeostasis. Mob Genet Elem 1: 274–278. 10.4161/mge.18301 22545238 PMC3337136

[14] Guzzardo PM, Muerdter F, Hannon GJ (2013) The piRNA pathway in flies: highlights and future directions. Curr Opin Genet Dev 23: 44–52. 10.1016/j.gde.2012.12.003 23317515 PMC3621807

[15] Wong LH (2010) Epigenetic regulation of telomere chromatin integrity in pluripotent embryonic stem cells. Epigenomics 2: 639–655. 10.2217/epi.10.49 22122049

[16] Silva-Sousa R, López-Panadès E, Piñeyro D, Casacuberta E (2012) The Chromosomal Proteins JIL-1 and Z4/Putzig Regulate the Telomeric Chromatin in Drosophila melanogaster. PLoS Genet 8: e1003153 10.1371/journal.pgen.1003153 23271984 PMC3521665

[17] Silva-Sousa R, Varela MD, Casacuberta E (2013) The Putzig partners DREF, TRF2 and KEN are involved in the regulation of the Drosophila telomere retrotransposons, HeT-A and TART. Mob DNA 4: 18 10.1186/1759-8753-4-18 23822164 PMC3726405

[18] Silva-Sousa R, Casacuberta E (2013) The JIL-1 kinase affects telomere expression in the different telomere domains of Drosophila. PLoS One 8: e81543 10.1371/journal.pone.0081543 24244743 PMC3828246

[19] Shpiz S, Kwon D, Rozovsky Y, Kalmykova A (2009) rasiRNA pathway controls antisense expression of Drosophila telomeric retrotransposons in the nucleus. Nucleic Acids Res 37: 268–278. 10.1093/nar/gkn960 19036789 PMC2615633

[20] Shpiz S, Olovnikov I, Sergeeva A, Lavrov S, Abramov Y, Savitsky M, et al (2011) Mechanism of the piRNA-mediated silencing of Drosophila telomeric retrotransposons. Nucleic Acids Res 39: 8703–8711. 10.1093/nar/gkr552 21764773 PMC3203600

[21] Piñeyro D, López-Panadès E, Lucena-Pérez M, Casacuberta E (2011) Transcriptional analysis of the HeT-A retrotransposon in mutant and wild type stocks reveals high sequence variability at Drosophila telomeres and other unusual features. BMC Genomics 12: 573 10.1186/1471-2164-12-573 22111838 PMC3235214

[22] Petit N, Piñeyro D, López-Panadès E, Casacuberta E, Navarro A (2012) HeT-A_pi1, a piRNA target sequence in the Drosophila telomeric retrotransposon HeT-A, is extremely conserved across copies and species. PLoS One 7. 10.1371/journal.pone.0037405 PMC3357415 22629389

[23] Siriaco GM, Cenci G, Haoudi A, Champion LE, Zhou C, Gatti M, et al (2002) Telomere elongation (Tel), a new mutation in Drosophila melanogaster that produces long telomeres. Genetics 160: 235–245. 11805059 10.1093/genetics/160.1.235 PMC1461955

[24] Schüpbach T, Wieschaus E (1991) Female sterile mutations on the second chromosome of Drosophila melanogaster. II. Mutations blocking oogenesis or altering egg morphology. Genetics 129: 1119–1136. 1783295 10.1093/genetics/129.4.1119 PMC1204776

[25] Bilder D, Haigo SL (2012) Expanding the Morphogenetic Repertoire: Perspectives from the Drosophila Egg. Dev Cell 22: 12–23. 10.1016/j.devcel.2011.12.003 22264728 PMC3266552

[26] Becalska AN, Gavis ER (2009) Lighting up mRNA localization in Drosophila oogenesis. Development 136: 2493–2503. 10.1242/dev.032391 19592573 PMC2709059

[27] Danilevskaya ON, Traverse KL, Hogan NC, DeBaryshe PG, Pardue ML (1999) The two Drosophila telomeric transposable elements have very different patterns of transcription. Mol Cell Biol 19: 873–881. 9858610 10.1128/mcb.19.1.873 PMC83944

[28] Casacuberta E, Pardue M-L (2002) Coevolution of the telomeric retrotransposons across Drosophila species. Genetics 161: 1113–1124. 12136015 10.1093/genetics/161.3.1113 PMC1462189

[29] Casacuberta E, Pardue M-L (2003) HeT-A elements in Drosophila virilis: retrotransposon telomeres are conserved across the Drosophila genus. Proc Natl Acad Sci U S A 100: 14091–14096. 14614149 10.1073/pnas.1936193100 PMC283551

[30] Wesolowska N, Amariei FL, Rong YS (2013) Clustering and protein dynamics of Drosophila melanogaster telomeres. Genetics 195: 381–391. 10.1534/genetics.113.155408 23893488 PMC3781967

[31] Zhang L, Beaucher M, Cheng Y, Rong YS (2014) Coordination of transposon expression with DNA replication in the targeting of telomeric retrotransposons in Drosophila. EMBO J 33: 1148–1158. 10.1002/embj.201386940 24733842 PMC4193921

[32] Wu X, Tanwar PS, Raftery LA (2008) Drosophila follicle cells: morphogenesis in an eggshell. Semin Cell Dev Biol 19: 271–282. 10.1016/j.semcdb.2008.01.004 18304845 PMC2430523

[33] Cenci G, Siriaco G, Raffa GD, Kellum R, Gatti M (2003) The Drosophila HOAP protein is required for telomere capping. Nat Cell Biol 5: 82–84. 10.1038/ncb902 12510197

[34] Savitsky M, Kwon D, Georgiev P, Kalmykova A, Gvozdev V (2006) Telomere elongation is under the control of the RNAi-based mechanism in the Drosophila germline. Genes Dev 20: 345–354. 10.1101/gad.370206 16452506 PMC1361705

[35] Chambeyron S, Popkova A, Payen-Groschêne G, Brun C, Laouini D, Pelisson A, et al (2008) piRNA-mediated nuclear accumulation of retrotransposon transcripts in the Drosophila female germline. Proc Natl Acad Sci U S A 105: 14964–14969. 10.1073/pnas.0805943105 18809914 PMC2567476

[36] Dufourt J, Dennis C, Boivin A, Gueguen N, Théron E, Goriaux C, et al (2014) Spatio-temporal requirements for transposable element piRNA-mediated silencing during Drosophila oogenesis. Nucleic Acids Res 42: 2512–2524. 10.1093/nar/gkt1184 24288375 PMC3936749

[37] Zhao Z, Pan X, Liu L, Liu N (2014) Telomere length maintenance, shortening, and lengthening. J Cell Physiol 229: 1323–1329. 10.1002/jcp.24537 24374808

[38] Pickett HA, Reddel RR (2012) The role of telomere trimming in normal telomere length dynamics. Cell Cycle 11: 1309–1315. 10.4161/cc.19632 22421147

[39] George JA, DeBaryshe PG, Traverse KL, Celniker SE, Pardue M-L (2006) Genomic organization of the Drosophila telomere retrotransposable elements. Genome Res 16: 1231–1240. 10.1101/gr.5348806 16963706 PMC1581432

[40] Homem CCF, Knoblich JA (2012) Drosophila neuroblasts: a model for stem cell biology. Development 139: 4297–4310. 10.1242/dev.080515 23132240

[41] George JA, Pardue M-L (2003) The promoter of the heterochromatic Drosophila telomeric retrotransposon, HeT-A, is active when moved into euchromatic locations. Genetics 163: 625–635. 12618401 10.1093/genetics/163.2.625 PMC1462444

[42] Ye J, Renault VM, Jamet K, Gilson E (2014) Transcriptional outcome of telomere signalling. Nat Rev Genet 15: 491–503. 10.1038/nrg3743 24913665

[43] Hrdlicková R, Nehyba J, Bose HR (2012) Alternatively spliced telomerase reverse transcriptase variants lacking telomerase activity stimulate cell proliferation. Mol Cell Biol 32: 4283–4296. 10.1128/MCB.00550-12 22907755 PMC3486134

[44] Montell DJ, Yoon WH, Starz-Gaiano M (2012) Group choreography: mechanisms orchestrating the collective movement of border cells. Nat Rev Mol Cell Biol 13: 631–645. 10.1038/nrm3433 23000794 PMC4099007

[45] Arbouzova NI, Bach EA, Zeidler MP (2006) Ken & barbie selectively regulates the expression of a subset of Jak/STAT pathway target genes. Curr Biol 16: 80–88. 10.1016/j.cub.2005.11.033 16401426

[46] Ovando-Roche P, Yu JSL, Testori S, Ho C, Cui W (2014) TRF2-Mediated Stabilization of hREST4 Is Critical for the Differentiation and Maintenance of Neural Progenitors. Stem Cells 32: 2111–2122. 10.1002/stem.1725 24740933

[47] Choi J, Southworth LK, Sarin KY, Venteicher AS, Ma W, Chang W, et al (2008) TERT promotes epithelial proliferation through transcriptional control of a Myc- and Wnt-related developmental program. PLoS Genet 4: e10 10.1371/journal.pgen.0040010 18208333 PMC2211538

[48] Veraksa A, Bauer A, Artavanis-Tsakonas S (2005) Analyzing protein complexes in Drosophila with tandem affinity purification-mass spectrometry. Dev Dyn 232: 827–834. 10.1002/dvdy.20272 15704125

