# Supplementary figures and images for: Specific Localization of the Drosophila Telomere Transposon Proteins and RNAs, Give Insight in Their Behavior, Control and Telomere Biology in This Organism
Source: PLoS One. 2015 Jun 12;10(6):e0128573. doi: 10.1371/journal.pone.0128573 (PMC4467039; doi:10.1371/journal.pone.0128573)

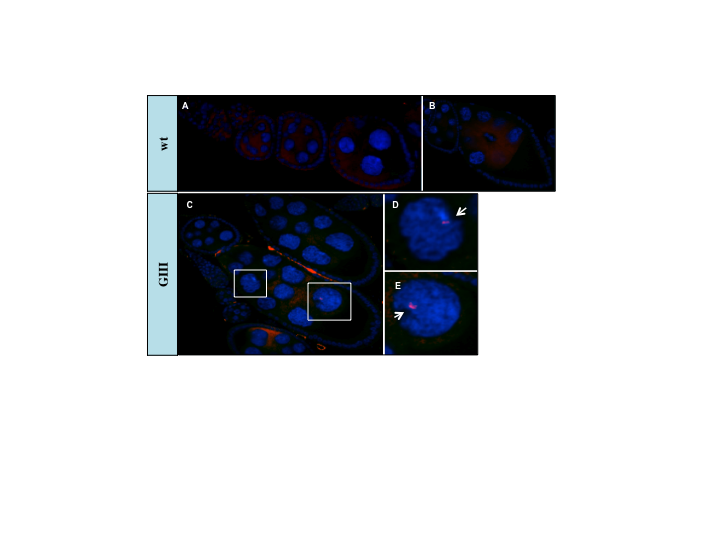

Supplement: S1 Fig — (TIFF) [file pone.0128573.s001.tiff]

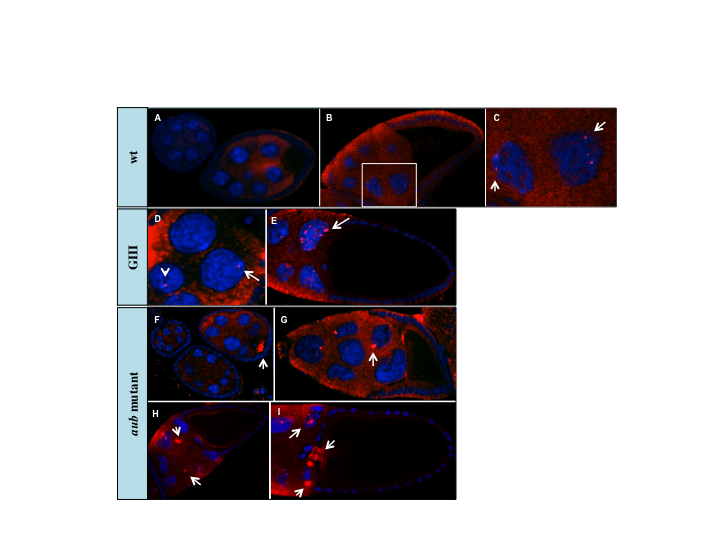

Supplement: S2 Fig — (TIFF) [file pone.0128573.s002.tiff]
